# Diseases of the Heart and Circulation

**Published:** 1904-07-23

**Authors:** 


### Progress in Medicine and Surgery.

### DISEASES OF THE HEART AND CIRCULATION.

**Dilatation of the Heart.**—Colbeck[1] is of opinion that all forms of cardiac dilatation have fundamentally a common mode of origin insomuch as they all depend primarily on an absolute or relative diminution or loss of the muscular tone of the organ. Under normal conditions the heart copes with heightened blood-pressure by an increased display of energy. Its work is largely determined by the degree of diastolic distension of the ventricle, which varies with the amount of blood that remains in the ventricular cavity after the completion of the systole. This diastolic distension is kept within physiological limits by the cardiac tonus, and as long as this is unimpaired the amount of residual blood is restricted. If the tone is diminished or lost, diastolic distension is not checked, the amount of residual blood is increased, and the chamber in contracting acts at a mechanical disadvantage. Dilatation therefore follows. The cardiac muscle has two properties, that of contraction and that of tonus, and the loss of the latter, though the former be unimpaired, will lead to dilatation. Tone is largely a matter of the nervous system, by which it is maintained and regulated. The beneficial operation of a cheerful disposition, the harmful effect of depression, are thus readily explained. In cases of valvular lesions, as long as the tone of the muscle fibre is maintained, dilatation does not occur, but it does so when the tonus is unable to provide sufficient elastic resistance to cope with the distensile stress of the excessive intra-ventricular pressure during diastole. In cases of heart failure without valvular lesion there are usually two main factors, either of which may occasionally act alone, an increase in the work required consequent on increased arterial resistance, and impairment of the muscular power of the organ, the result of structural disease of the cardiac walls, or of nutritive neuro-muscular or other disturbances.

**Rheumatic Carditis and Pericarditis.**—The treatment of these conditions is discussed in the first of the Harveian lectures by Lees.[2] Salicylates are as truly antirheumatic as mercury is an antisyphilitic; and this is true not merely of rheumatic arthritis but of carditis and pericarditis as well. Under its administration the general condition improves, the cardiac state is ameliorated, the area of dulness diminishes, and the strength of the ventricular contraction increases. The drug should be given in adequate doses, and its use must not be too soon relinquished. Children require proportionately larger doses than adults, and fortunately bear them well, rarely having deafness, tinnitus, headache, or vomiting. Salicylates may exceptionally produce a dangerous symptom—a marked deepening of the inspiration, resembling that of diabetic coma. This should immediately lead to a discontinuance of the drug. But it is always advisable to give with each dose of the salicylate double the quantity of sodium bicarbonate; if this is done air-hunger will never take place. Salicylates are sometimes credited with being depressing, especially to the heart. But observation of patients with rheumatism, who have not taken salicylates, shows that the poison of rheumatism, like that of diphtheria and influenza, weakens and causes dilatation of the heart. The cardiac dulness may extend to the left, to one or even two finger-breadths beyond the nipple line. Experiments of Gaskell showed that lactic acid causes dilatation and finally diastolic standstill of the frog's heart, whilst a dilute solution of sodium hydrate produces exactly the opposite result. This suggests that the rheumatic poison is of an acid character. And it has been shown by Walker and Ryffel that the micrococcus rheumaticus produces formic acid in considerable quantity, with another acid probably acetic; this can be also obtained from the bodies of the micro-organisms, and from the urine of rheumatic fever patients. The micrococcus also has a marked hæmolytic action on red blood corpuscles, and in cultures produces an albumose which produces marked pyrexia. These observations are a justification for the administration of large doses of bicarbonates with the salicylate. The cardiac dilatation of rheumatism rarely if ever produces fatal syncope, unlike diphtheria and influenza, but it may give rise to obvious pallor and changes in the pulse. As regards local treatment of rheumatic carditis and pericarditis, leeches and an ice-bag may be used. Leeches may be applied over the cardiac area, but are better put over the lower anterior part of the right chest. They lessen the supply of blood through the azygos vein, and thus relieve distension of the right auricle. Normally the dulness of the right auricle extends about one finger-breadth to the right of the sternum in the fourth space. This dull area may increase to two finger-breadths and extend into the third space when the right auricle is distended. This may occur in mitral disease, in acute pulmonary disease, such as pneumonia and acute bronchitis. In the case of rheumatism it is the left ventricle which is primarily affected, as above stated, but in pericarditis the right auricle may dilate: the symptom of this is hurried breathing, rapid and deep inspirations, and the physical sign is an increase of dulness in the fourth right space to two finger-breadths. In such a case leeches, or better still, venesection (4 to 8 oz.) will be found to give great relief. It may be well to follow this by hypodermic injection of strychnine. Meanwhile, hot-water bottles should be applied to the patient's lower limbs and afterwards an ice-bag should be placed over the precordium. This should be re-filled every two hours and the hot-water bottles every three hours. It is found that the above treatment checks the inflammation, increases the vigour of the muscular fibre and diminishes the dilatation, thus enormously assisting the forces which make for repair.

**Enlargements of the Heart.**—In a lecture on this subject Hale White[3] says that if symptoms of heart failure occur in association with adherent pericardium, either the valves of the heart are diseased, or that organ is enlarged, or its muscular substance is diseased, or the adhesions have strangled the large veins as they enter the heart. The pericardium is of value as a support, it prevents the over-distension of the heart which would otherwise occur during severe muscular exertion. If inflamed the pericardium is weakened, and the heart may dilate, and dilatation soon leads to hypertrophy. If adhesions form during the stage of dilatation they will tend to prevent the heart contracting. It is therefore of importance in treating pericarditis to secure, if possible, that adhesions shall take place when the heart is not dilated. The patient should be kept in horizontal position in bed, constipation should be avoided, and digitalis should be given to contract the heart. In the dilated heart of specific fevers, *e.g.* scarlet fever, pneumonia, influenza, and diphtheria, strychnine is of great service; it may be given in the following prescription: 5 minims of liquor strychninæ, 15 minims of tincture of digitalis, with 5 grains of caffein and $2\frac{1}{2}$ grains of salicylate of sodium to ensure the solution of the caffein, to an ounce of water. A distinct form of cardiac enlargement is that due to alcohol. The symptoms are that the patient, always a hard drinker, is very short of breath, and has a rapid feeble pulse, often 150 per minute. The area of cardiac dulness is increased; the impulse is diffuse, feeble, displaced outwards; the cardiac sounds are very weak, but as a rule no murmur is present, the second sound may be reduplicated. It is surprising how ill such patients may be before any signs of backward pressure show themselves, and they may die before they are seen by a physician. Both sides of the heart are hypertrophied, but as a rule no important histological changes can be seen, though occasionally patches of interstitial inflammation may be found scattered through the heart. The causes of this alcoholic dilatation and hypertrophy are obscure. Neuritis of the vagus is one theory, hydræmic plethora due to the large quantities drunk is another, but no satisfactory explanation has as yet been offered. The diagnosis is fairly easy. Valvular mischief and chronic Bright's disease must be first excluded. Acute myocarditis always follows a specific fever, and chronic myocarditis is rare and does not cause a rapid pulse or great dyspnœa. Fatty degeneration is usually associated with general anæmia, wasting diseases as phthisis or cancer, or over-eating. Fibroid disease of the heart is of importance, because it is often a cause of sudden and unexpected death. The change is seldom seen externally, consisting usually of a fibroid degeneration in the interventricular septum. The left ventricle is hypertrophied, the increase depending on the degree to which the fibrous material impairs the working of the heart. The cause of the fibrosis is uncertain; in some cases it may be syphilis, but this cause will explain only a certain portion, perhaps a quarter or a third of the cases.

[1] Lancet, April 9. [2] Brit. Med. Jour., Nov. 21. [3] Clin. Jour., Oct. 28.

*(To be continued.)*

